# Antifungal Effect of Non-Woven Textiles Containing Polyhexamethylene Biguanide with Sophorolipid: A Potential Method for Tinea Pedis Prevention

**DOI:** 10.3390/healthcare2020183

**Published:** 2014-04-08

**Authors:** Hiromi Sanada, Gojiro Nakagami, Kimie Takehara, Taichi Goto, Nanase Ishii, Satoshi Yoshida, Mizuyuki Ryu, Yuichiro Tsunemi

**Affiliations:** 1 Department of Gerontological Nursing/Wound Care Management, Graduate School of Medicine, The University of Tokyo, 7-3-1 Hongo, Bunkyo-ku, Tokyo 113-0033, Japan; E-Mails: gojiron-tky@umin.ac.jp (G.N.); gottai-tky@umin.ac.jp (T.G.); yoshida-s@saraya.com (S.Y.); 2 Department of Nursing Administration, Graduate School of Medicine, The University of Tokyo, 7-3-1 Hongo, Bunkyo-ku, Tokyo 113-0033, Japan; E-Mail: kwaku-tky@umin.ac.jp; 3 Biochemical Laboratory, Saraya Co., Ltd., 24-12 Tamatecho, Kashiwara, Osaka 582-0028, Japan; E-Mails: ishii-n@saraya.com (N.I.); ryu@saraya.com (M.R.); 4 Promote Development Division, Saraya Co., Ltd., 2-2-8 Yuzato, Higashisumiyoshi-ku, Osaka City, Osaka 546-0013, Japan; 5 Department of Dermatology, Tokyo Women’s Medical University, 8-1 Kawada-cho, Shinjuku-ku, Tokyo 162-8666, Japan; E-Mail: ytsun-tky@umin.ac.jp

**Keywords:** fungal adherence, fungal infection, permeability, self-care, tinea pedis

## Abstract

Tinea pedis is a preventable skin disease common in elderly or diabetic patients. Daily foot washing is effective for prevention, but can be difficult for many patients. Additionally, conventional methods cannot eliminate fungi within the stratum corneum, a common site for fungal invasion. This study investigates the antifungal effects, cytotoxicity, permeability, and efficacy of non-woven textiles containing polyhexamethylene biguanide (PHMB) mixed with sophorolipid. Permeability of PHMB with varying concentrations of sophorolipid was assessed via a cultured skin model. Stratum corneum PHMB concentration was quantified by polyvinylsulphuric acid potassium salt titration and cytotoxicity was assayed via 3-(4,5-dimethylthiazol-2-yl)-2,5-diphenyltetrazolium bromide. Antifungal effects were evaluated via a new cultured skin/*Trichophyton* *mentagrophytes* model, with varying PHMB exposure duration. Clinically-isolated Trichophyton were applied to the feet of four healthy volunteers and then immediately treated with the following methods: washing with soap, a non-woven textile with PHMB, the textile without PHMB, or without washing. Fungal colony forming units (CFUs) were evaluated after one of these treatments were performed. Sophorolipid with various concentrations significantly facilitated PHMB permeation into the stratum corneum, which was not in a dose-dependent manner. Significant PHMB antifungal effects were achieved at 30 min, with low cytotoxicity. Textiles containing PHMB significantly reduced CFU of fungi in healthy volunteers to levels comparable to soap washing. Our results indicate the utility of this product for tinea pedis prevention in clinical settings.

## 1. Introduction

Tinea pedis is one of the fungal infections caused by dermatophytes [1]. *Trichophyton rubrum* and *T. mentagrophytes* account for over 90% of causative fungi. The prevalence of tinea pedis increases with age, with the highest prevalence among those 50 to 60 years of age [2]. The main nutrient of dermatophytes is keratin, which is located in the stratum corneum. These fungi often cause skin maceration and erosion between the toes, leading to secondary bacterial infection. This is critical to elderly and diabetic patients, as secondary infections cause foot ulcers, cellulitis, necrotizing fasciitis, or gas gangrene, making prevention of infection most important [3].

Since dermatophytes invade the keratin layer within 24 h after attachment, daily foot washing is recommended for efficient physical removal of fungi [4,5]. Previous reports indicate foot washing using soap can eliminate fungi from the skin surface [6]. However, daily foot washing can be difficult for many patients, owing to limited bathing of residents in long-term care facilities, limited joint range of motion, or visual impairment [7,8]. Furthermore, the skin barrier function of the stratum corneum also makes dermatophyte removal within the keratin layer difficult using only conventional antiseptics [9,10]. Therefore, a clinically effective, simple, and easy-to-use method is needed.

To overcome these challenges, we developed a new non-woven textile product soaked with an antifungal agent with enhanced permeability to the stratum corneum. This product can physically eliminate fungi attached to the skin surface and interferes with growth in the stratum corneum via application to the skin surface. This study investigates its effect on antifungal function *in vitro* and *in vivo* in healthy volunteers.

## 2. Experimental

### 2.1. Evaluation of Permeation of PHMB with Sophorolipid Treatment within the Skin Model

Polyhexamethylene biguanide (PHMB; Arch UK Biocides Ltd., Blackley, UK) alone or with 0.1 or 1% sophorolipid (synthesized as previously described [11]) was added to the top of the stratum corneum of a three-dimensional cultured human skin model (LSE-high, Toyobo Co., Ltd., Osaka, Japan) and quantified in the stratum corneum after 120 min to determine the degree of permeation. PHMB was extracted from homogenized stratum corneum sample of each well in 1 mL of phosphate-buffered saline and filtered. For quantitation, each sample was mixed with sodium chloride (1 N) adjusted to a pH of 1.5 ± 0.05, then titrated with a standardized aqueous solution of polyvinylsulphuric acid potassium salt (N/400, Wako Pure Chemical Industries, Ltd., Osaka, Japan) to a blue to pink toluidine blue indicator color change endpoint (*n* = 3).

### 2.2. MTT Assay for Cytotoxicity

To assess cytotoxicity, the cultured skin model was treated with 0.1% PHMB with 0.1% sophorolipid supplementation for 5, 30, 60, and 120 min, and then incubated with 3-(4,5-dimethylthiazol-2-yl)-2, 5-diphenyltetrazolium bromide (MTT; Sigma-Aldrich, Tokyo, Japan). Yellow tetrazolium salt is reduced by mitochondrial enzymes in viable cells to an insoluble blue formazan product. The skin model without PHMB was used as a control. After incubation, samples were transferred to new 1.5 mL tubes and mixed with 200 µL of isopropanol to extract any resulting formazan. Absorbance was measured spectrophotometrically using an automated microplate reader (Spectra Thermo, Tecan Group Ltd., San Jose, CA, USA) at a wavelength of 570 nm (*n* = 3). Cell survival was computed by using the following formula: Cell viability % = [(mean optical density of the sample − blank)/(mean optical density of the control − blank)] × 100.

### 2.3. Evaluation of Antifungal Effect: In Vitro

We established an intra-stratum corneum *T. mentagrophytes* model to evaluate the antifungal effect of 0.1% of PHMB. *T. mentagrophytes* was incubated at 30 °C on 3.9% autoclaved potato dextrose agar (Nissui Pharmaceutical Co., Tokyo, Japan) slant medium for a week. The slant medium was then mixed with 10 mL of 0.05% Tween80. Conidia were collected by centrifugation at 3,000 rpm for 5 min after removal of fungal filaments by filtration, washed with 0.05% Tween80-normal saline solution three times, then suspended into 4 mL of 0.05% Tween80 for fungal quantification. To mimic infected skin, *T. mentagrophytes* was inoculated onto the surface of the cultured skin model at 10^5^ colony forming units (CFUs)/cm^2^, then incubated for 7 days at 37 °C under 5% CO_2_.

To evaluate the antifungal capacity, a filter paper (Finn Chambers, φ8 mm, SmartPractice^®^, Phoenix, AZ, USA) impregnated with 50 µL of 0.1% PHMB with 0.1% sophorolipid supplementation was placed onto cultured skin (LabCyte EPI-MODEL 24, Japan Tissue Engineering Co., Gamagori, Japan) inoculated with *T. mentagrophytes* for 7 days and incubated for 5, 30, 60, and 120 min at 37 °C under 5% CO_2_. After incubation with 0.1% PHMB with 0.1% sophorolipid, cultured skin was immediately homogenized and fungi was extracted. CFUs of fungi were counted via plate culture (*n* = 3). The inoculated cultured skin without PHMB treatment was used as baseline (Time = 0).

### 2.4. Evaluation of Antifungal Effect: In Vivo

We recruited four healthy volunteers free from tinea pedis and immunosuppression. To confirm whether the volunteers are free from tinea pedis, visual skin inspection was performed by a trained researcher and the absence of dermatophyte was tested by culture method. These were challenged for 5 s with clinically isolated Trichophyton by placing a foot onto paper soaked with 60 mL of normal saline containing one streak of Trichophyton cultured on an agar plate. This procedure was repeated for four times within the same individual. The participants then immediately washed their feet in one of three ways: with soap, using a non-woven textile impregnated with PHMB and 0.1% sophorolipid, or using a non-woven textile soaked in tap water. In the other way the feet were remained unwashed as a control. Fungal CFUs were counted using the previously described foot-press method [12]. All study protocols were approved by an institutional review board (approval number (#3411-(1)), the risks thoroughly explained, and all participants provided written informed consent. To avoid fungal infection, the volume of challenged fungi was restricted and the foot skin was thoroughly washed after completion of experiments.

### 2.5. Statistical Analysis

Data are presented by means and standard deviations. Multiple comparison among the groups for PHMB permeation analysis was done with Bonferroni adjustment. Time course analyses for cytotoxicity and antifungal effect were done by one-factor repeated analysis of variance using the baseline data (Time = 0) as a control. A mixed effects model was used to test the effect of various washing method on the log-transformed CFU for the healthy volunteer experiment. Fixed effect was washing method and a random effect was participant with a compound symmetry structure. Differences among the washing methods in terms of log-transformed CFUs were assessed by multiple comparison with Bonferroni adjustment. P values less than 0.05 was considered statistical significant. All statistical analyses were performed using Statistical Analysis System 9.3 (SAS Institute, Cary, NC, USA).

## 3. Results and Discussion

**Figure 1:**
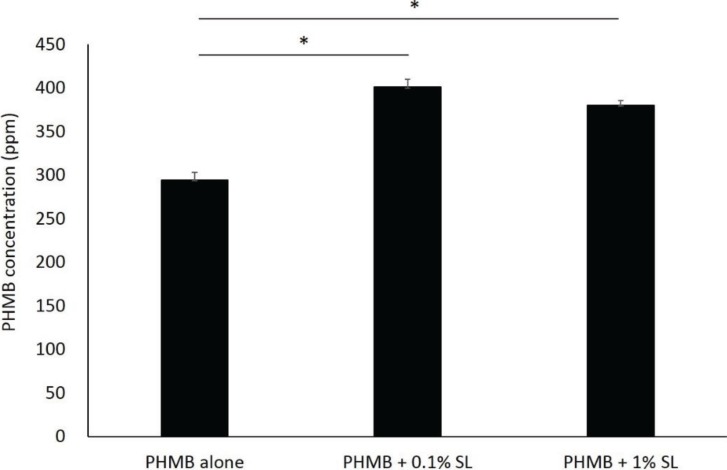
Permeability of polyhexamethylene biguanide (PHMB) into stratum corneum. Permeability was assessed using a cultured skin model. PHMB was added to the culture medium with a final concentration of 0.1%, with 0.1 and 1% of sophorolipid, then incubatedfor 120 min. Inner stratum corneum PHMB concentration was quantified via polyvinylsulphuric acid potassium salt titration. Data represent mean with SD (*n* = 3). * *p* < 0.001.

**Figure 2:**
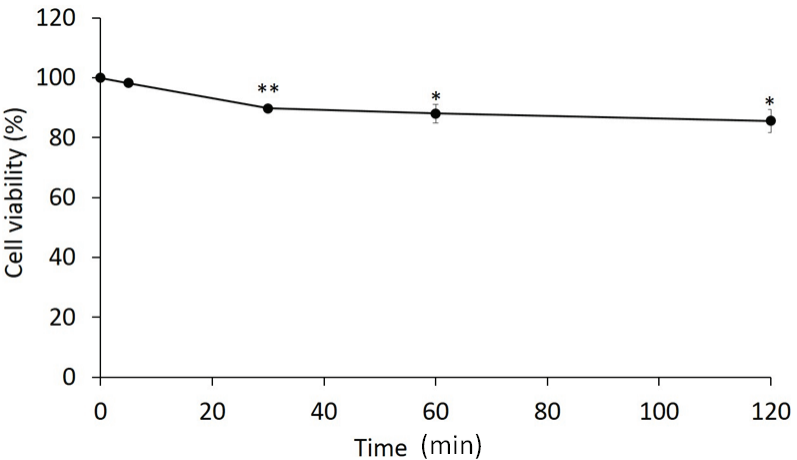
Cytotoxicity of PHMB with sophorolipid. An MTT assay was used to determine cytotoxicity. Cell viability% = [(mean optical density of the sample − blank)/(mean optical density of the control − blank)] × 100. Data represent mean with SD (*n* = 3). * *p* < 0.01, ** *p* < 0.001, compared to the baseline value.

**Figure 3:**
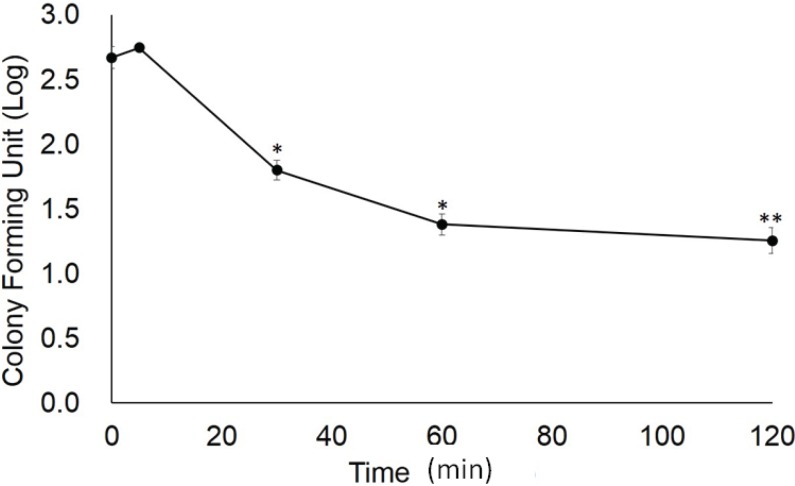
Antifungal effect of PHMB with sophorolipid *in vitro*. Values indicate log-transformed colony forming units (CFUs). CFUs of fungi within each cultured tissue sample was determined by agar plate culture. Data represent mean with SD (*n* = 3). * *p* < 0.01, ** *p* < 0.001, compared to the baseline value.

Adding sophorolipid significantly increased the concentration of PHMB within the skin (*p* < 0.001 for both sophorolipid supplemented groups compared to PHMB alone, Figure 1); however, at the two sophorolipid concentrations tested there were no significant differences in the final concentration of PHMB (*p* = 0.053). Cell viability was significantly decreased to 85.7% after 120 min of exposure of the mixture (*p* = 0.023 compared to the baseline, Figure 2). The *in vitro* infection model demonstrated that PHMB with sophorolipid supplementation decreased number of fungi to less than one-tenth after 1-hour exposure, similar to a 2-hour exposure (Figure 3).

**Figure 4:**
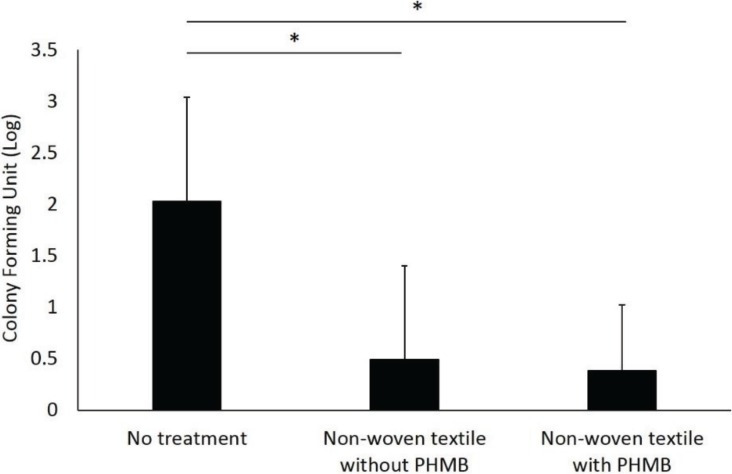
Antifungal effect of PHMB in healthy volunteers. Values indicate log-transformed CFUs. To calculate log CFUs for each feet, we added 0.5 for each raw value. Value in the soap washing group was zero and not shown in this graph. Error bars indicate standard deviation (*n* = 4). * *p* < 0.001, multiple comparison by Bonferroni adjustment.

According to the *in vitro* experiments, we confirmed the promising effectiveness for inhibiting the growth of *T. mentagrophytes* within stratum corneum, therefore we proceed to the healthy volunteer experiments. The mean age of the four participants was 32.8 ± 11.3 years, with three females (75%) and one male in the volunteer experiment. The mean number of CFUs in the non-treatment group was 765.5 (Figure 4). Soap washing completely eliminated attached fungi from the foot surface (*p* < 0.001 compared to the non-treatment group). The non-woven textiles with PHMB decreased the number of fungi to a mean of 5.8 CFUs and the textiles without PHMB to a mean of 18.9 CFU (*p* < 0.001, *p* < 0.001, respectively, compared to the non-treatment group). The CFUs in the non-woven textiles without PHMB was significantly higher than the soap washing groups (*p* = 0.041). However, there was no significant difference between the non-woven textiles with PHMB and the soap washing group. There were no adverse events reported.

In this study, we sought to determine the effectiveness of a non-woven textile containing PHMB supplemented with sophorolipid on fungal activity *in vitro* and *in vivo*.

PHMB is a commercially available general biocide that disrupts the cell membranes of microorganisms, causing leakage of intracellular components and inhibiting respiratory enzymes [13]. This is widely used as a swimming pool and contact lens disinfectant, and is also used for antimicrobial wound dressings to control infection [14,15]. The cytotoxicity assay revealed only 20% cell death at 2 h exposure, which is less than other major antiseptic agents. Müller *et al*. reported PHMB is a biocompatible agent with high efficiency for bactericidal activity and low cytotoxicity when compared with other agents such as chlorhexidine digluconate, povidone iodine in ointment, or sulphadiazine, which are commonly used in the clinical setting [16]. Reduced cytotoxicity is fundamental to the underlying keratinocytes which must be preserved. PHMB appears to have an acceptable level of both cytotoxicity and antifungal activity.

Supplementation with sophorolipid facilitated the permeation of PHMB into the stratum corneum. Sophorolipid is a glycolipid-type biosurfactant, which has been reported to enhance transdermal delivery of active ingredients by forming assemblies [11]. There was no dose-dependent relationship between the sophorolipid concentration and PHMB concentration, indicating a sufficient effect was achieved with 0.1% supplementation.

After determining the efficacy of PHMB for eliminating fungi with low cytotoxicity, we then investigated its effect on eliminating dermatophytes attached to the foot in healthy volunteers. This protocol successfully yielded high numbers of dermatophytes on agar plates when the feet remained unwashed and complete elimination by soap washing. The non-woven textiles with PHMB considerably decreased fungal counts, but so did the textiles without PHMB, albeit with slightly higher CFU counts. It is noteworthy that the fungal count in the non-woven textiles without PHMB was significantly higher than in the soap washing group and the significant difference was not seen in the non-woven textiles with PHMB and the soap washing comparison. This suggests physical removal of fungi by scrubbing the skin surface plays a major role in fungal clearance, but PHMB may contribute to the additional reduction [6]. Since the non-woven textiles with PHMB possess the anti-fungal ability as demonstrated in the present study, it is expected that the number of rubbing the foot skin would be less for using this textiles than for soap washing, which might facilitate the individuals with limited joint range of motion and access to the bathing care to perform daily foot care by themselves. Further study is needed to prove the antifungal effectiveness of PHMB on human skin and ultimately in the patients with tinea pedis.

A major limitation of this study is assessing the antifungal effect immediately after fungi inoculation. As fungi invade the stratum corneum after a certain period of attachment in the clinical setting [4], antifungal effects would ideally be assessed at a later phase. Additionally, the effectiveness in preventing tinea pedis should be tested in a long-term interventional trial. PHMB may have a more significant effect in patients with stratum corneum infiltration, as opposed to surface colonization. As Hammer *et al.* reported that dermatophyte susceptibility varies towards antimicrobial textiles, we need to consider the further experiments against different species even though they indicated *T. mentagrophytes* growth remained unaffected in direct contact with the antimicrobial textiles tested [17].

Although there are many products available for washing feet, they are not designed to prevent tinea pedis among patients with limited physical capacity. We hope to enhance patient self-care by designing easy-to-use sheet-type materials with high antifungal effectiveness and low cytotoxicity. This would be suitable for daily use by patients for whom daily foot washing is difficult.

## 4. Conclusions

Our results indicate the non-woven textiles with containing PHMB with sophorolipid favorable reduction of Trichophyton *in vitro* and *in vivo*, suggesting potential clinical effectiveness for tinea pedis prevention.
